# Annual Meeting of Presidents at St. James's Palace

**Published:** 1914-12-26

**Authors:** 


### THE LEAGUE OF MERCY.

### Annual Meeting of Presidents at St. James's Palace.

The fifteenth annual meeting of Presidents of the League of Mercy was held at St. James's Palace on Thursday, December 17. The chair was occupied by the Right Honourable the Lord Farquhar, G.C.V.O., and supporting him on the dais was H.R.H. Princess Alexander of Teck, the Lady Grand President of the League. There was a large attendance, and during the proceedings the Lady Grand President presented the Order of Mercy to the ladies and gentlemen whose names had been sanctioned for this distinction by H.M. the King, the Sovereign of the Order.

### The Chairman said he felt sure that all present would deeply regret the unavoidable absence of the Grand President. As was well known, his Serene Highness Prince Alexander of Teck was serving the country with the Expeditionary Force, and, while all missed him, they were all proud of him. The League was fortunate in having present H.R.H. the Lady Grand President. All would know and appreciate the benevolence of H.R.H. the Duchess of Albany, whose absence on this occasion the members would deplore. The Council had hoped for the presence of H.H. the Princess Victoria of Schleswig-Holstein, one of the most active supporters of the League in the home counties, but owing to an obligation to attend another appointment she had found it impossible to be present, to their great regret.

The League of Mercy and the War.

At the last annual meeting of the League, the Grand President suggested that more attention should be paid to the organisation, and, with this idea, he suggested that in the efficient districts more members and more collectors should be secured. And just at the time when this very excellent suggestion was made, and when the result was coming in satisfactorily, in August last this terrible war came unexpectedly upon us. With this event some very necessary and excellent funds were brought into existence which it was thought might compete, in an unsatisfactory manner, with the League of Mercy. The point at once came up for consideration by the Grand President and Council, and the result of their deliberate consideration was the issue of a most excellent circular urging all the vice-presidents, members, and collectors to use all their energies to increase the funds, and to do all in their power to support the extra strain which had come upon the hospitals which received sick and wounded soldiers for treatment. He was sure her Royal Highness would support the Council in rendering their most grateful thanks to all those who so readily responded to the circular, and, if he might venture, he would ask them to redouble their efforts during the present year. As a distinguished statesman said to him, this was a time not for speeches, but for action, and so his own speech would be a short one. He urged upon this company interested in the League, who were as numerous as usual, to remember those words, that this is a time for action.

The Year's Collections.

He did not wish on the present occasion to go much into details, but desired to point out that this year the League had been lucky enough to receive nearly £18,000—a very large sum—which made a total since the inception of the League of £250,000. That achievement all might very well be proud of. There had been a difficulty in getting the figures for the year, but he believed a sum of £14,000 would be given to King Edward's Fund. Another interesting and most satisfactory feature was that the extra-Metropolitan hospitals would receive £2,356, being an increase over the amount granted last year, and the highest sum yet so distributed. Another point on which the Hon. Treasurer was kind enough to write to him this morning was that the Distribution Committee would consider what it was possible to do during the year in that most excellent cause of supporting the hospitals which were taking in for treatment sick and wounded soldiers. He desired also to thank all those members of the League who so kindly gave entertainments during the year. Amongst others he would specially mention the garden-party given by Lord and Lady Michelham, to which every single member of the League received an invitation. He also wished to utter a word of thanks to his distinguished friend Lord Midleton, who, in the midst of his enormous number of engagements, had been kind enough to come this afternoon, and they would have the benefit of an address from him.

Congratulations from the King and Queen.

He had just had placed in his hands a letter which Mr. Wallington had sent at the command of His Majesty the King: "The King and Queen continue to take the deepest interest in the progress and work of the League of Mercy, and it is a great satisfaction to their Majesties to hear that, in spite of the difficulties which have been experienced this year, the amount collected will even exceed the total realised last year. The King and Queen heartily congratulate the Presidents and the Lady Presidents and those who have worked with them on this successful result."

### Viscount Hill moved: "That the Right Hon. the Lord Farquhar, G.C.V.O., be appointed Chairman, Sir Frederick Green, J.P., Deputy-Chairman, and Captain A. E. Speer Hon. Secretary respectively for the annual meeting of Presidents for the ensuing year." He did not think any words of his were necessary to bring these gentlemen to the notice of members for their re-election. All would realise the great and valuable work they had done for the League in the past, and those present, as workers in the good cause, could not do better than re-elect them for another year. The League deserved congratulation on the very able work it had done during the past year, which had been a very difficult one for such efforts. But everybody had been most willing; some had doubled and some trebled their gifts in his locality. He felt sure that if only the League were kept before people, perhaps more than had been done in the past, though next year might be an even more difficult one, its return might exceed the present satisfactory figure.

### Alderman Sir Charles Wakefield moved that seven representative members of the Executive Committee be appointed:—The Right Hon. the Lord Farquhar, G.C.V.O., Sir George Alexander, LL.D., J.P., Sir Frederick Green, J.P., Mr. Edward Murray Ind, D.L., J.P., the Hon. Mrs. George Lawson Johnston, the Dowager Lady Dimsdale, and Mrs. R. L. Harrison. He said he was sure all would agree that these ladies and gentlemen had placed their time and influence cheerfully at the disposal of the League of Mercy, and he had great pleasure in submitting the resolution, which was seconded by Mr. H. W. Keay and carried.

### Sir Henry Burdett

(Hon. Treasurer) said 1914 had been the most difficult year that the League had experienced. And he welcomed those difficulties, because they had proved emphatically that the work of the League was full of life, that its principles were sound, and that it must continue to live after the present workers had disappeared. It was a wonderful thing to him, as well as a gratifying point, that in looking through all the returns, whether they were smaller or larger, there was evidence to show that good work had been done everywhere; that the workers had done their utmost to maintain the position of the League. The total number of districts working in 1914 was ninety-four —including a new branch which had been started in India—as against ninety-two in the previous year. The average approximate yield from each district in 1914 was £197, as against £189, the actual yield in 1913. The approximate total collections for 1914 were £18,482, making an increase for 1914 of £1,138. As the Chairman had already said, the grants made to the extra-Metropolitan hospitals were £2,356, an increase of £38 on the amount of the grants in the previous year. The total amount distributed by the League of Mercy in its fifteen years of work was: to extra-Metropolitan hospitals, £16,740; and to the King's Fund to the year 1913, £202,000. For 1914 it was hoped that the same grant could be made as last year—namely, £14,000, which would make the total amount distributed by the League since its inception £232,740, a sum which had been raised without cost, and given freely to the hospitals by this League during its fifteen years of activity. He considered this a splendid record. He had specially to mention a most seasonable and welcome gift. Sir William Dunn, on his decease, left a large sum of money at discretion for distribution, and, by Lady Dunn's wish, a communication was sent by Sir William's executors to say that they had great pleasure in making a grant of £1,500 to the League of Mercy, and sending a cheque at once, because they were sure the money would be welcome. That came at the lean period of the year, just before the outbreak of the war. It saved the League considerable expense, and everyone would feel immensely indebted to Lady Dunn for her forethought.

#### Good Work in Every District.

It was very difficult for him (the speaker) to know what to do in referring to the amounts which had been collected in each district. Sometimes the Treasurer's remarks were thought to be too long because he had too many lists and too many details; sometimes people were pleased when their names were mentioned; some did not like their names to be mentioned. As he had to try to meet the wishes of the 16,000 concerned in the League it was not easy to know what to do. He, therefore, had made a new departure in posting near the door a list of those districts which had contributed in the order of the amounts, so that all interested could see what had been done. All he proposed to do in his speech was to mention two record collections and, for the rest, emphasise the whole-hearted and continuous labours of everybody in the League during 1914. The first record was that in East Sussex, where the sum raised in 1914 was £441. This district commenced in 1904 with 5s. In the following year this was increased to two guineas, and now this year the collections had reached £441. He was mentioning these facts as an encouragement. Another instance was a district which had had many experiences, and where there was a great and prosperous voluntary hospital, so that there hospital work should be fully appreciated—namely, Croydon. From Croydon the League received in 1914 £231, the largest sum raised since the work began there. It started with a small contribution; in 1899 the collection was £64, and for several years it was about that figure. This year, on account of the zealous work of the President, Mr. W. J. Chamberlain, the Lady President, the Duchess of Albany, and the Hon. Secretary, Miss Hall, the splendid maximum of £231 had been reached. And in connection with the East Sussex Branch he wished specially to mention the energy of the President, Viscount Hythe; the Lady President, Lady Idina Hythe; and the Hon. Secretary, Mr. J. A. Paton. He would also specially congratulate the Deptford and Brockley Division, which had gone up from £121 to £193 this year; and Romford, which had advanced from £115 to £162. Colchester showed an increase from £48 to £90, and in Camberwell, where they had had rather a curious history, because in 1900 they sent £117, in 1913 it fell to £62; the figure this year was £84. He therefore hoped Camberwell was in a fair way to develop in the same way as East Sussex had.

He had mentioned these instances—representing rich boroughs like Croydon, poor boroughs like Camberwell and Deptford, suburban places like Romford, and county divisions like Colchester and East Sussex—to show that everywhere the spirit of the League was strong enough, if it were only understood, to carry the work forward and cause the sums given to the hospitals through the League to be greater, even in a year like this, than they had been before.

#### A Special Grant for Hospitals Treating the Wounded.

He did not wish to take up the time of the meeting, as he agreed with the remark of the statesman friend of the Chairman; but he wanted to explain that there was a wish that some extra distribution could be made to those hospitals which had admitted to their wards the war's sick and wounded. But circumstances varied in the different hospitals; the Government made grants to certain hospitals, though not necessarily to all of them, and the grants varied from 2s. to 4s. per diem.

It would be apparent, therefore, that the League had not yet been able to get the necessary details to enable such a distribution to be made before the end of this year. It was hoped, however, that the idea would commend itself to those present, and that in every district of the League the work done by the hospitals for the wounded would be brought forward in the beginning of 1915, and that the matter would not be deferred until October. If this were done, the Executive promised they would ask for a return about May, to ascertain what had been accomplished by that date. If the special efforts had been successful, the Council hoped to be able to make a special grant by the end of June to those hospitals which had been taking in wounded soldiers. In that way the total contribution for the year might be increased, and at the same time the good work of the League in this special direction would be enhanced.

#### The Voluntary Hospitals Brigade.

One feature which should be a great help this year was the universal wish which was evident on the part of everybody, especially the ladies, to do something to help in this war. The cause of the sick and wounded was immensely helped by people joining the League and giving their time to collecting small sums of money.

Lord Kitchener had asked, and was continually asking, for "more men," more recruits for the period of the war. He (the speaker) would like also to ask for recruits, especially women—women who would join the League for the period of the war, and make it their business, however humble they might be, to raise £1 each for each year the war lasted, to go towards the expense of providing for the sick and wounded in our hospitals. Our voluntary hospitals had done gloriously : directly the war broke out they gave of their very best, gave their physicians, surgeons, nurses, and staff generally, to help in the work necessitated by the war. And when the Territorial system had to be organised it was the voluntary establishments which came forward and helped the organisation, each in its own district; they gave generously in order to make the work a success. He knew that all present were proud of the voluntary hospitals. And those who knew most about the work which had been done this year, the amount of self-sacrifice and self-denial and energy represented in its accomplishment, felt an added pride in the voluntary system, and a reverence for the work. The suggestion has been made that there should be a Voluntary Hospitals Brigade, so that those who joined the League for the war could be members of that Brigade, each one of them undertaking to raise at least £1, mostly, he hoped, in very small sums. That idea had already been tested in a tentative way, for in one week one hundred people in various parts of the country had spontaneously offered to join such a Brigade. Such willingness, he thought, must ultimately contribute to helping forward the League.

He desired to thank all warmly and gratefully for the work they had done. He had never felt, in all his forty-eight years of work, more encouraged and more grateful as treasurer than when stating this year what returns had come in. These returns meant that the League was strong and helpful and was going forward, that it was a strong and indispensable aid to the sick and wounded throughout the country. He would leave his hearers with that thought, in the hope that they might see their way to begin working in January for the sick and wounded, and that the Council would have the happiness to hear from them, some time in May, that the extra effort had succeeded, and that, therefore, the League would be justified in making special grants to those hospitals who had come forward to treat the sick and wounded from the war.

### Sir William Collins said he did not think there was need for him to say very much on behalf of the Honorary Secretaries; but the contrast could hardly be greater between the circumstances and conditions which confronted the League this time last year and those which prevailed now. In the time of peace last year the League of Mercy had to chronicle a falling revenue, and were apprehensive lest a system of National Insurance might imperil the very existence of voluntary hospitals altogether, and be an introduction to a system of State or Municipal hospitals.

Vindication of the Voluntary System.

But to-day, on the contrary, in time of war, they had the pleasure to record a rising revenue for the League; and he thought they had the right to claim a most emphatic vindication in time of war of the voluntary hospital system; for in times of peace we were beginning to learn that even with a system of National Insurance and an army of panel doctors there was a frequent and almost as great a resort to voluntary hospitals in cases of doubt, difficulty, and in cases requiring serious operations, as ever before. So in time of war, after the medical departments of the State had done their best—and no one would disparage them—an appeal was made to the voluntary hospitals, to those who were trained nurses and to doctors in the voluntary hospitals, that they would give their services; and all were aware of the splendid and abundant response which had been made to that appeal, not only for the relief of the British sick and wounded, and for those of our Allies, but even for the sick and wounded of the enemy. It was his privilege, in a recent visit to Flanders, to see not only in and around Dunkirk, but also in Furnes, the convents adapted as improvised hospitals, and the staff and nurses, some of them trained in London hospitals, ministering to the wounded under great difficulties. And out of Nieuport and along the Yser canal he saw motor ambulances worked by those who had been trained in voluntary hospitals, ministering, under the most perilous and trying conditions, to the sick and wounded in the battlefield. Therefore, he thought it could be claimed, in times both of peace and war, that the State felt the limitation of its own system, and gladly resorted to voluntary hospitals from which to recruit the staffs and to deal with the most difficult cases which came under their observation. It was not without significance that the nation which had been able to show the most satisfactory system of dealing with the sick and wounded in war was that nation which alone had adhered to the voluntary system.

The League's Workers.

With regard to the management of the League and the speeding-up of its work, as advocated by his Serene Highness last year, during the past year Vice-Presidents to the number of 129 had been added, and, putting against that the forty-nine who had resigned, there was a net gain of eighty. Unfortunately, there was one topic which never failed them : the record of loss by death. These included the Duke of Argyll, Lord Clarendon, Lord Strathcona, and General Sir George Barker. Testimony had been made to the work in some of the poorer districts. There were at any rate two districts in which penny collections had been made at intervals; 2,786 separate penny collections had been made by Miss Meadows at Camberwell, and 5,879 separate penny collections made by Mr. Berry, of the Strand district. That indicated not only that the small subscriber was doing his share, but it also showed the arduous character of the work in some of the branches. He believed the League would go forward in the year to come and secure that additional support which the noble Chairman desired.

[Viscount Midleton then delivered an address, which is reported on p. 283.]

### Sir George Alexander moved a vote of thanks to Lord Midleton for his address. His Lordship had referred to the great success of the address of Father Vaughan last year, but he, Lord Midleton, had made an equally good score. The results of the League's working in 1914 must be very gratifying, both to the Chairman and to the Lady Grand President. The League of Mercy possessed a title of immense value in peace time, and still more so in time of war; and if they spread that one magic word "Mercy" through the land, they might feel sure that 1915 would be a still better year than the present one.

**Mr. E. Murray Ind** seconded the vote, remarking that all had listened with great interest to Lord Midleton's address on hospitals and healing. He paid a high tribute to the work of the collectors, especially in country districts. With some there had been a slight falling off in honorary members, and perhaps they were a class whom it was easiest to switch off from one charity to another.

**Mr. W. J. Chamberlain** moved a vote of thanks to the Right Hon. the Lord Farquhar, and expressed thanks for the kindly words which had been spoken about Croydon. Sometimes in the provinces collectors were asked why residents were expected to subscribe to London hospitals when they had a local hospital; and the answer was that the London hospitals received difficult and critical cases from all parts; and he referred to that argument because the same might be said in other districts.

**The Rev. R. Howel Brown** seconded, adding a word of personal thanks to Colonel Kempster and those working with him for all they had done, and for the sympathetic reception they gave to all callers.

**The Chairman,** in reply, said the League of Mercy had always interested him very much; but during the last two years he had lost his best men in Marylebone. Still, he hoped to show a better result next year.

### Recipients of the Order of Mercy.

Presidents.—The Lord Michelham, K.C.V.O. (W. St. Pancras), Mr. E. Murray Ind (Mid-Essex).

Lady Presidents.—The Honble. Mrs. Wood of Hongrave (Stowmarket), Lady Alexander (S. St. Pancras), Mrs. H. H. Clutton (E. St. Pancras), Mrs. Godding (Poplar-Limehouse), Mrs. Pigeon (Brighton-Hove), Mrs. Price (Maldon), Mrs. Hornby Steer (Lambeth-Kennington).

Vice-Presidents.—Sir Horace Brooks Marshall (S.E. Essex), Alderman J. Francis (S.E. Essex), Mr. L. L. Franks (Hackney North).

Lady Vice-Presidents.—The Countess of Cassillis (St. George's, Hanover Square), The Honble. Rosamund Bateman Hanbury (Eye), The Honble. Mrs. Mark Hovell (W. St. Pancras), The Honble. Mrs. Cecil Wilson (Horsham), Lady Cooper (Hants), Lady Furley (N. Kensington), Miss Abraham (Hornsey-Finchley), Mrs. Cuthbert Brereton (Brentford), Mlle. Dessaint (S. Kensington), Mrs. Ellis (Hants), Mrs. Gosling (Berks), Mrs. Myles Kennedy (S. Kensington), Miss Lloyd (Fulham), Miss M. Jessie Lovell (St. Pancras North), Miss Lucas (Hitchin), Miss Maude Meadows (Camberwell), Mrs. H. W. Nightingale (Wimbledon), Mrs. Stuart H. Rickman (Berks), Mrs. Ronalds (Chelsea), Mrs. Rowcliffe (Guildford), Mrs. G. Pearce Sparrow (Epsom-Esher), Mrs. Tonge (Stowmarket), Mrs. Vine (Epsom-Esher).

Members.—Mr. E. Brown (Uxbridge), Mr. H. Crighton (Woolwich), Mr. F. J. Huxtable (Marylebone East), Mr. C. Randall (St. Pancras North), Mr. Augustus H. Sprange (S. Paddington), Mr. E. R. Townsend (Strand), Mr. A. G. Warren (Kingston-Surbiton), The Honble. Mrs. Charles White (Hants), Miss F. Adcock (Hampstead), Mrs. J. H. Bere (Fulham), Mrs. Charles Cave (Hammersmith), Miss M. E. Clarkson (Deptford-Brockley), Mrs. Coley (S. Kensington), Miss Edmonds (St. Pancras North), Mrs. Frost (Deptford-Brockley), Mrs. Graves (Bucks), Miss Edith Hopkins (Fulham), Mrs. F. Leney (S. Paddington), Miss H. Litten (Paddington South), Mrs. May (Deptford-Brockley), Miss Misa (N.E. Sussex), Mrs. George Simpson (Reigate), Mrs. Stoddart (Mid-Essex), Miss Tompkins (St. Pancras West), Miss Tuck (Chelsea), Mrs. Voller (Battersea), Mrs. Waring (Westminster).

